# Growth Arrest Specific Gene 6 Protein Concentration in Cerebrospinal Fluid Correlates with Relapse Severity in Multiple Sclerosis

**DOI:** 10.1155/2013/406483

**Published:** 2013-05-27

**Authors:** P. P. Sainaghi, L. Collimedaglia, F. Alciato, R. Molinari, D. Sola, E. Ranza, P. Naldi, F. Monaco, M. Leone, M. Pirisi, G. C. Avanzi

**Affiliations:** ^1^AOU “Maggiore della Carità,” 28100 Novara, Italy; ^2^(Interdisciplinary Research Center of Autoimmune Diseases) IRCAD, Via Solaroli 17, 28100 Novara, Italy; ^3^Department of Translational Medicine, “A. Avogadro” University, 28100 Novara, Italy

## Abstract

*Background*. Growth arrest specific gene 6 (Gas6) protein enhances survival of oligodendrocytes and neurons, and it is involved in autoimmunity. Therefore, we aimed to verify whether cerebrospinal-fluid (CSF) Gas6 concentration may represent a biomarker of disease activity in multiple sclerosis. *Methods*. Sixty-five patients who underwent a spinal tap during relapse of relapsing/remitting multiple sclerosis (RR-MS)(McDonald-criteria) were studied. Forty patients affected by noninflammatory/nonautoimmune neurological diseases served as controls. Relapse was defined according to Schumacher criteria. Symptoms were grouped according to Kurtzke-Functional System (FS). Clinical characteristics of the relapse, duration, Expanded-Disability-Status Scale (EDSS) change, number of FS involved, completeness of recovery, age, steroid therapy, were categorised. Patients were followed at 6-month intervals to assess relapse rate and EDSS progression. Gas6 was measured (CSF, plasma) by in-house-enzyme-linked immunoassay (ELISA). *Results*. Higher CSF Gas6 concentrations were observed in relapses lasting ≤60 days (8.7 ± 3.9 ng/mL) versus >60 days (6.5 ± 2.6) or controls (6.5 ± 2.4; *P* = 0.05), with ≤2 FS involved (8.5 ± 3.8) versus >2 FS (5.6 ± 2.5) (*P* < 0.05) and EDSS change ≤2.5 points (8.8 ± 3.7) versus >2.5 (6.5 ± 3.5) (*P* = 0.04). Conversely, CSF Gas6 was not predictive of the completeness of recovery. Plasma and CSF concentrations were not related (*R* ^2^ = 0.0003), and neither were predictive of relapse rate or EDSS progression after first relapse. *Conclusions*. CSF concentration of Gas6 is inversely correlated with the severity of relapse in RR-MS patients but does not predict the subsequent course of the disease.

## 1. Introduction

Multiple sclerosis (MS) is an immune-mediated disorder of the central nervous system (CNS) determined by an inflammatory aggression to the myelin of neuronal fibers. The pathogenesis of MS involves complex interactions between many cell types, including both the adaptive and innate immune systems. In this context, activated macrophages and microglial cells mediate myelin degradation and oligodendrocyte damage by producing proinflammatory cytokines such as tumor necrosis factor- (TNF-) alpha and interferon-gamma [1]. For this reason, molecules involved in dampening of macrophages activation could be involved in MS pathogenesis.

Gas6 (growth arrest specific 6), a multimodular secreted protein, binds and activates receptors belonging to the Tyro-3 tyrosine kinase receptor family (Tyro-3, Axl, and Mer) also known as TAM receptors [2–5]. Gas6/TAM interaction is involved in several physiological processes, including cell migration, adhesion, cell growth, and survival [6]. Recently, it has been claimed that Gas6/TAM system may be involved in the pathogenesis of autoimmune diseases [7]. This hypothesis is based on the observation that knock-out mice for TAM receptors display aberrant proliferation of T and B lymphocytes, with diffuse tissue infiltration and autoimmune manifestations including vasculitis, lupus-like lesions, and autoantibodies. This phenotype is likely the consequence of monocytes-macrophages hyperactivity [8]. The importance of Gas6 in controlling the immune response is supported by the finding that Gas6 inhibits macrophage activation and cytokine production, reducing interleukin (IL)-6 and TNF-alpha secretion through the activation of Mer receptors [9].

Direct evidences link Gas6 to autoimmune processes within the central nervous system (CNS). Gas6 and its TAM receptors are expressed in the brain of embryonic and adult rats proportionally to synaptogenesis of cerebral tissues [10–12]. In addition, Gas6 promotes proliferative and antiapoptotic effects on several CNS cell types as hippocampal and cortical neurons [11, 13], Schwann cells [14] and, in particular, oligodendrocytes, which are protected from apoptosis trough Axl/PI3-K/Akt activation [15, 16]. Binder and colleagues have also shown that the absence of Gas6 affects the efficiency of remyelination following a demyelinating insult, induced by cuprizone and that defect of remyelination is accompanied to reduction in the number of mature oligodendrocytes [17]; moreover the administration of recombinant human Gas6 to the corpus callosum enhanced the myelin repair in the same mouse model [18]. These observations suggest that the Gas6/TAM system may be involved both in the regulation of the survival of neuronal and glial cells, in particular of those involved in myelination, and the control of the innate immune response, two paramount processes in MS pathogenesis. Based on these premises, in the present study we measured CSF and plasma Gas6 protein during a relapse of multiple sclerosis in relation to relapse clinical features and severity scores as Kurtzke-Functional-System (FS), to verify their usefulness as a biomarker of disease course.

## 2. Methods

### 2.1. Patients

Sixty-five consecutive patients admitted to an academic referral centre from 2001 to 2005 with a diagnosis of clinically isolated syndrome (CIS) or Relapsing-Remitting (RR) MS were studied. CIS was defined as the occurrence of an acute or subacute event of the CNS affecting the optic nerves, brainstem, or spinal cord of presumed inflammatory demyelinating origin in a patient with no history suggestive of an earlier demyelinating episode [19]. McDonald criteria [20] were used to diagnose MS. Inclusion criteria were age ≥18 years, first visit to the MS Centre, diagnostic lumbar puncture during the relapse, no treatment with corticosteroids prior to the lumbar puncture, and no prior treatment with any immunomodulant drug. Patients with primary progressive MS were excluded. The majority of patients (*n* = 45) were sampled during their first relapse; the others were observed and sampled at their second (*n* = 16) or third relapse (*n* = 4); moreover at sampling 22 were CIS and 43 RR-MS. After the first visit, 55 patients (10 patients were lost to follow-up) were prospectively followed and visited thereafter every 6 months; the follow-up ended on July 31, 2009 (Table 1). Follow-up duration ranged from 2.5 to 11 years (mean 5.9 years; SD 1.8). At the end of this follow-up period, the diagnosis was CIS in 9 cases and RR-MS in 42, while 4 patients had already entered secondary progression.

The study was approved by a local Ethical Committee and conducted in strict accordance with principles of the Declaration of Helsinki. Each patient gave a written informed consent before the lumbar puncture to the use of his/her CSF for experimental evaluation and to maintain data in the data-base.

### 2.2. Relapse Definition and Evaluation

Relapse was defined as “acute or subacute occurrence, recurrence or worsening of symptoms of neurologic dysfunction attributable to MS, lasting more than 24 hours after a period of at least 30 days of improvement or stability” [21]. Neurological deterioration of preexisting symptoms accompanied by fever was not considered a relapse. Paroxysmal episodes were considered as relapse when multiple and occurring over not less than 24 hours. Symptoms occurring within a month after the initial symptoms of relapse were considered to be part of the same episode. Patients data were collected at the first visit at the centre during the relapse and also at 3, 7, and 30 days after the relapse onset. For each relapse, type of symptoms, signs, and dates of occurrence were collected, as previously described [22]. Briefly, symptoms and signs were grouped to fit in with the Kurtzke Functional System (FS) [23]. The score for each FS and the total EDSS score were calculated at onset, at maximum worsening (zenith) and at the first examination after the day of maximum improvement of the relapse. We correlated the following factors with Gas6 concentration: relapse duration and severity, recovery, gender, age at the onset of the relapse, number of affected FSs, Link index (≤0.70/>0.70), annualized relapse rate, and annualized EDSS progression. The relapse duration was calculated as the time between the date of onset of the first symptom and the date of maximum improvement of the last symptom. The severity of the relapse was calculated as the difference between the EDSS score at the day of maximum worsening and the EDSS score before the onset of the relapse; the lowest ΔEDSS was 0.5 (1.0 for the first relapse). Recovery was evaluated at the date of maximum improvement and classified as complete (EDSS = 0) or incomplete (EDSS ≥ 1). Annualized relapse rate was calculated as the number of relapses during the follow-up divided by the months of the follow-up. Annualized EDSS progression was calculated as the difference between the EDSS score of the last follow-up visit available and the EDSS score after the first relapse, divided by the years of follow-up.

### 2.3. CSF and Plasma Sampling and Gas6 Measurement

CSF samples of MS patients were drawn within 90 days from the symptoms onset and before complete resolution of the relapse; 29 patients underwent also a blood draw in the same day of the spinal puncture. CSF and plasma samples were obtained from a biobank including patients affected by a noninflammatory/nonautoimmune neurological diseases (NINAD) such as ischemic stroke, amyotrophic lateral sclerosis, headache, and psychiatric conditions simulating neurological diseases or otologic dizziness. CSF and plasma samples of cases and controls were stored at −30°C. Gas6 was measured by a sandwich ELISA developed and validated in our laboratory for human plasma samples [24, 25] and with the same method modified for CSF assay as previously described [26]. Samples from MS patients had been collected between 2001 and 2005, appropriately aliquoted and stored at −80°C to be tested for Gas6 concentration between 2009 and 2010. Samples from NINAD controls were also collected along the years from 2001 and 2005, processed and stored similarly, and used for Gas6 testing in 2007; these latter data had already been published [26]. Briefly a 96-well plate (NUNC ImmunoPlates MaxiSorp F96, NUNC, Hereford, UK) is coated overnight with anti-human-Gas6 primary antibody (goat polyclonal affinity purified IgG, R&D Systems, Minneapolis, MN. USA). The antigen is detected by a secondary biotin conjugated antibody (Biotinylated anti-human Gas6 antibody, R&D Systems, Minneapolis, MN. USA), a streptavidin-peroxidase conjugate (Sigma, St. Louis, MO, USA), and TMB (3,3′,5,5′-tetramethylbenzidine, Sigma, St. Louis, MO, USA). The reaction is blocked with sulphuric acid 1.8 and absorbance detected at 450 nm with a reference wavelength set at 570 nm. Optical density is fitted versus nominal concentration by applying a four-parameter logistic regression to the calibration curve prepared in Gas6 depleted plasma or in BSA (Bovine serum albumin, further purified fraction V, ≥98%, Sigma, St. Louis, MO, USA). The method has been validated according to the Food and Drug Administration guidelines for inter- and intra-assay % coefficient of variation (%CV) for Gas6 measurement in human plasma and CSF (all %CVs were within 15% with negligible matrix effect). There was no cross-reactivity with human protein S (MP Biomedicals, Solon, OH, USA). The lowest quantification limit was 0.26 ng/mL [24–26].

### 2.4. Statistical Analysis

Data were collected and stored in an electronic database to be analyzed with the Statistica statistical software program, release 10 (StatSoft, Tulsa, OK, United States). The Shapiro-Wilk test was performed to assess normality for any continuous variable analyzed. The measures of central tendency and dispersion used throughout the paper were means and standard deviations for continuous variables with normal distribution. Accordingly, normally distributed variables were compared among groups by means of the Student's *t-*test, ANOVA, and post hoc comparisons of the means (Tukey's post hoc test for nonhomogeneous samples/test of Spjotvoll-Stoline). Categorical variables were analyzed with Pearson chi-square test and stepwise logistic regression to identify independent predictive factors of CSF Gas6 variations. A level of 0.05 (two-sided) was chosen to indicate statistical significance.

## 3. Results

**Figure 1:**
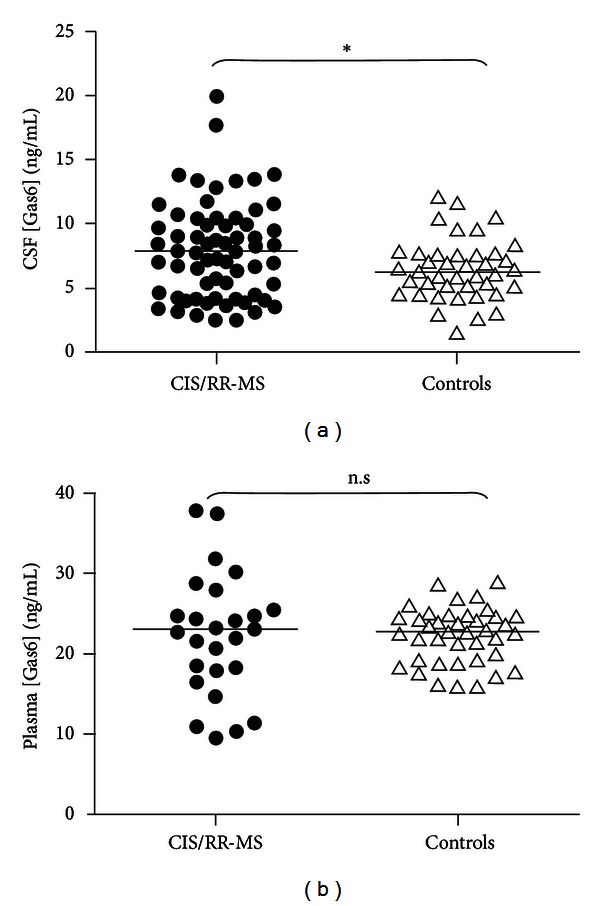
Gas6 concentration in CSF of patients with MS. (a) The scatter plot displays CSF Gas6 concentrations of patients with MS (black circles) with respect to controls (white triangles). *indicates *P* < 0.05 in statistical comparison of the means (Student's *t* test); the horizontal bar indicated the mean value. (b) The scatter plot displays plasma Gas6 concentrations of patients with MS (black circles) with respect to controls (white triangles). The horizontal bar indicated the mean value.

Mean CSF Gas6 concentration was nearly equal in CIS with respect to RR-MS patients (7.8 ± 3.2 ng/mL versus 7.9 ± 3.8, Student's *t*-test, *P* = n.s.) but significantly higher in CIS/RR-MS (*n* = 65; 37 females) patients with respect to controls (*n* = 40; 22 females) (7.9 ± 3.7 ng/mL versus 6.5 ± 2.4 ng/mL resp., *P* < 0.03) while plasma Gas6 concentration was not significantly different between groups (Student's *t* test, *P* = n.s.) (Figure 1).

**Figure 2:**
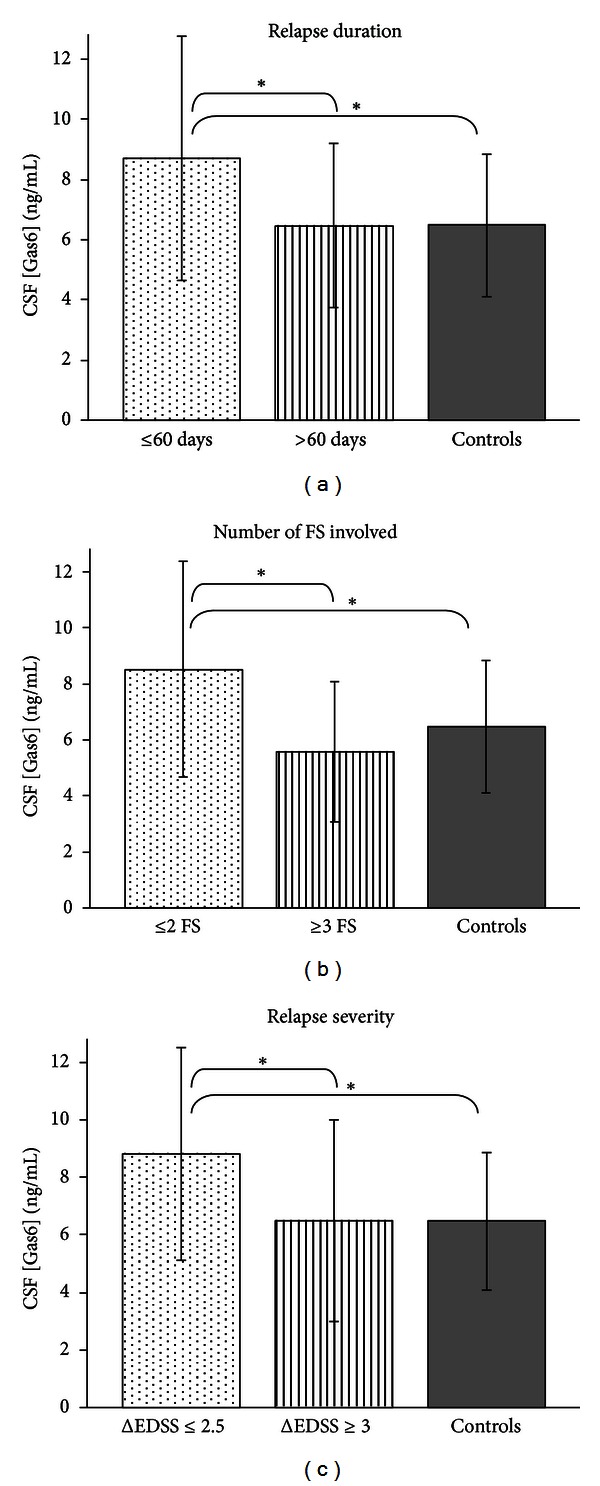
Gas6 concentration in CSF of patients with MS in relation to relapse features. The graph shows means and standard deviations of CSF Gas6 concentrations in relation to relapse duration: ≤60 days (dots), >60 days (vertical lines) and controls (gray) (panel a); number of FS involved during the relapse: ≤2 (dots), ≥3 (vertical lines) and controls (gray) (panel b); and EDSS score variation at maximum worsening (severity): ≤2.5 (dots), ≥3 (vertical lines) and controls (gray) (panel c). *indicates *P* ≤ 0.05 in statistical comparison of the means (Tukey's post hoc test).

**Figure 3:**
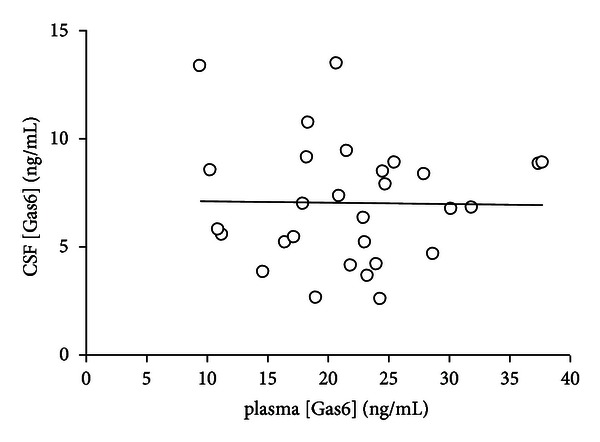
Dissociation between CSF and plasma Gas6 concentration. The figure displays the absence of correlation between CSF and plasma Gas6 concentration measured simultaneously during the first relapse in MS patients. The line indicates the regression equation: CSF Gas6 = 0.007 · plasma Gas6 + 7.1, *r* ^2^ = 0.0003, *P* = ns.

Figure 2 shows mean CSF Gas6 concentration in relationship to the duration of relapse. CSF Gas6 concentration was significantly higher in shorter relapses (relapses lasting ≤60 days 8.7 ± 3.9 ng/mL, versus >60 days 6.5 ± 2.6 ng/mL and controls 6.5 ± 2.4 ng/mL; ANOVA: *F* = 5.8, *P* < 0.004; Tukey post hoc test, *P* = 0.05), in relapses with fewer FS involved (≤2 FS 8.5 ± 3.8 ng/mL versus ≥3 FS 5.6 ± 2.5 ng/mL versus controls; ANOVA: *F* = 6.7  *P* < 0.002; Tukey post hoc test, *P* < 0.05) and in relapses with lower severity (ΔEDSS ≤ 2.5 8.8 ± 3.7 ng/mL versus ≥3 6.5 ± 3.5 ng/mL versus controls; ANOVA: *F* = 6.6   *P* < 0.002; Tukey post hoc test, *P* < 0,04). In particular, CSF Gas6 concentration was inversely proportional to the EDSS score of the relapse (*R* ^2^ = 0.11, *P* < 0.01) especially in pyramidal or cerebellar ones (*R* ^2^ = 0,25, *P* < 0,003). On the other hand, CSF Gas6 concentration did not change according to completeness of recovery, being similar in patients with or without complete recovery (8.2 ± 4.0 ng/mL versus 7.6 ± 3.5 versus controls, ANOVA, *P* = n.s). Alternatively, CSF Gas6 did not vary according to age, therapy, Link index (*P* = n.s.). Conversely, plasma Gas6 concentration was not related to any of the clinical variables listed above (ANOVA, *P* = n.s). Indeed, there was no relation between CSF and plasma Gas6 concentration (*R* ^2^ = 0.0003, *P* = n.s), as shown in Figure 3. In stepwise logistic regression, relapse duration and number of FS involved were significantly associated with Gas6 concentration (*F* : 13.3 and 6.5 resp., *P* < 0.02). Treatment allocation according to relapse features is shown in Table 2.

Follow-up data for a minimum of 2.5 years were available for 55 out of 65 patients and are presented in Table 3. Gas6 concentration did not differ according to annualized relapse rate either in CSF (Student's *t* test: ≥0.25/year 8.4 ± 4.6 versus <0.25/year 8.0 ± 3.2 ng/mL, *P* = n.s.) or in plasma (21.5 ± 6.4 versus 21.3 ± 12.4, *P* = n.s) and according to annualized EDSS progression either in CSF (≥0.2 EDSS points/year 7.9 ± 3.1 versus <0.2 8.1 ± 4.8, *P* = n.s.) or in plasma (21.8 ± 8.8 versus 23.4 ± 7.6 ng/mL, *P* = n.s). Moreover, neither CSF nor plasma Gas6 concentration correlated to annualized relapse rate or EDSS progression in linear regression analysis (data not shown, *P* = n.s.).

## 4. Discussion

The present paper shows that CSF Gas6 concentration is slightly higher in patients suffering a first relapse of MS than in control patients. Interestingly, a significant and substantial increase in CSF was observed in patients suffering from shorter, less severe, and less FS involved relapses. Conversely, patients who suffered from more severe relapse did not show any variations of CSF Gas6 concentration with respect to control subjects. This apparent paradox is reconciliated by considering Gas6 as a protective protein, able to limit inflammatory demyelination. This hypothesis is sustained by several in vitro and in vivo studies that indicate for Gas6 a role as inhibitor of macrophages activation [7, 9] and as a survival factor for neurons and oligodendrocytes [11, 15, 16]. Indeed, Gas6 knock-out mice challenged with cuprizone display increased demyelination damage with greater lesions and a higher number of apoptotic oligodendrocytes, infiltrating macrophages and microglial cells [6]. In absence of Gas6 there is a delay in remyelination of the lesions induced by cuprizone [17]. Moreover, Gas6 concentration is raised in the CSF of patients suffering from CIDP, a chronic autoimmune demyelinating disease of the peripheral nerves which shares some similarities with MS [26]. In summary, our results fit well the data in the literature, suggesting that MS patients with higher CSF Gas6 might be able to limit the extent of inflammatory demyelination. On the other hand Gas6 elevation in CSF seems to be related to relapse features rather than to the presence of MS itself. Additionally, since an altered Gas6/TAM ratio has been evidenced in established MS lesions [27], an impaired expression of TAM-receptor in absence of alteration of Gas6 concentration may concurr to influence the disease course. Further studies are needed to address this hypothesis. 

The mechanisms by which Gas6 might exert this putative protection by the effects of inflammatory demyelination remain speculative. Gas6 could act on macrophages and/or glial cells; this hypothesis is supported by the observations indicating that Gas6 downregulates the production of proinflammatory cytokines, such as IL-6 and TNF-alpha, and enhances the phagocytosis of cellular debris and apoptotic bodies through Mer receptor by activated macrophages, dendritic and microglial cells [7, 9, 28–30]. Thus, Gas6 may be able to limit the activation of the innate immune response and, consequently, the aggression to myelin fibers favoring cell debris and apoptotic bodies clearance which are, in turn, inflammatory triggers. Since mononuclear activation with cytokine production is crucial in the amplification of the autoimmune aggression to myelin and the failure to clear apoptotic cells is widely considered a trigger of autoimmunity, Gas6 may have an important role in this context. Alternatively, Gas6/TAM interaction could be relevant in processes of recovery after inflammatory demyelination, since it is well demonstrated that it enhances the survival of oligodendrocytes and neurons [11, 15], as happening for other cell lines [31], and mice lacking Gas6 have demyelinating lesions with a higher proportion of apoptotic oligodendrocytes during cuprizone-induced demyelination [6]. Consequently, Gas6 may participate actively in oligodendrocyte survival after myelin damage favoring a prompt repair. It is conceivable that patients who suffer from more severe relapse and who do not present an increase in CSF Gas6 concentration may have a defect in Gas6 expression and, consequently, present a defect in mechanisms of self-limitation to immune aggression to CNS or in myelin damage repair. It must be pointed out, however, that we were unable to identify a relation between Gas6 concentration in CSF and lack of complete recovery after the relapse, which could be considered the clinical correlate of an efficient recovery system.

The putative role of Gas6 in the control of immune aggression in the nervous system is strengthened by the finding that plasma and CSF Gas6 concentration are unrelated in MS patients; therefore, the increase of Gas6 in the CSF was not dependent on an increased plasma concentration and/or the breakdown of the blood-brain barrier, but likely on production by inflammatory cells within the CNS. These results are in line with previous results from our group concerning autoimmune polyneuropathies [26, 32]. Moreover, the relation between CSF Gas6 and EDSS score was stricter in pyramidal and cerebellar relapses, possibly because EDSS is more sensitive to the damage extension in pyramidal and cerebellar relapses with respect to other symptoms that are mainly subjective and less easy to score.

Although the sample size was small and only a few patients had an active disease with a significant number of relapses or severe disability progression, our data suggest that measuring Gas6 concentration in CSF of MS patients can only be useful to predict the evolution of a first relapse but not the subsequent disease course. Thus, Gas6 may be important in limiting the acute inflammatory demyelination during a relapse, but less than so in the pathogenic mechanisms that determine relapse recurrences and cumulative disability. 

In conclusion, CSF Gas6 protein concentration is significantly increased in milder MS relapses, suggesting an involvement of this protein in limiting autoimmune demyelinating processes or in favouring myelin repair after damage. Gas6 measurement in CSF during a relapse can be useful to predict how a relapse will evolve, but not the disease course in the long term. Further studies are warranted to clarify the Gas6 and TAM receptors role in the pathogenesis of MS-relapses and its clinical applications.

## Figures and Tables

**Table 1:** Features of the population studied.

Population studied	Number of patients
First relapse/second/third relapse	45/16/4
CIS/RR-MS	22/43
Patients with follow-up ≥2.5 years	55

CIS: clinically isolated syndrome; RR-MS: relapsing remitting multiple sclerosis.

**Table 2:** Treatment allocation according to relapse features.

Relapse features	Type of treatment			*χ* ^2^ analysis
	No	Oral PDN*	IVMP**	
Duration ≤60/>60 days	22/10	7/5	16/5	1.15, *P* = n.s.
Number of FS involved (≤2/>2)	26/7	8/4	16/4	0.88, *P* = n.s.
Severity, ΔEDSS (≤2.5/>2.5)	22/11	7/5	11/9	0.78, *P* = n.s.

PDN: prednisone, IVMP: intravenous methylprednisone, FS: functional system, and ΔEDSS: variation from baseline of Expanded-Disability-Status Scale.

*Oral prednisone was administered at 50 mg/day for the first 5 days then 25 mg/day for the following 5 days and 12.5 mg/day for further 5 days and then discontinued.

**Intra venous methylprednisone was administered at 1000 mg/day i.v. infusion for 5 consecutive days.

**Table 3:** Follow-up cohort features.

	M ± SD	Median
Follow-up duration (years)	5.9 ± 1.8	5.2
Annualized relapse rate (relapse/year)	0.32 ± 0.25	0.25
Annualized relapse progression (ΔEDSS/year)	1.6 ± 1.0	1.0
